# A Nutrigenomic Approach to Non-Alcoholic Fatty Liver Disease

**DOI:** 10.3390/ijms18071534

**Published:** 2017-07-16

**Authors:** Paola Dongiovanni, Luca Valenti

**Affiliations:** 1 Internal Medicine and Metabolic Diseases, Fondazione IRCCS Ca’ Granda Ospedale Policlinico Milano, Milan 20122, Italy; 2 Department of Pathophysiology and Transplantation, Università degli Studi di Milano, Milan 20122, Italy

**Keywords:** nonalcoholic fatty liver disease, nutrigenomics, dietary nutrients, genetic factors

## Abstract

Following the epidemics of obesity due to the consumption of high-calorie diet and sedentary lifestyle, nonalcoholic fatty liver disease (NAFLD) is now the leading cause of liver disease in Western countries. NAFLD is epidemiologically associated with metabolic syndrome and insulin resistance, and in susceptible individuals it may progress to cirrhosis and hepatocellular carcinoma. Genetic factors play a key role in NAFLD predisposition by interacting with nutritional and other environmental factors. To date, there is no drug therapy for the treatment of NAFLD, and the main clinical recommendation is lifestyle modification. In the last years, nutrigenomics is promoting an increased understanding of how nutrition affects the switch from health to disease by altering the expression of an individual’s genetic makeup. The present review tries to summarize the most recent data evidencing how the interactions between nutrients and genetic factors can influence NAFLD development. The final goal should be to develop tools to quantify these complex interactions. The definition of a “nutrigenomic risk score” for each individual may represent a novel therapeutic approach for the management of NAFLD patients.

## 1. Introduction

Nonalcoholic fatty liver disease (NAFLD) is defined by fat accumulation exceeding 5% of liver weight not explained by at-risk alcohol intake [1]. NAFLD is epidemiologically associated with metabolic syndrome and insulin resistance (IR) [2,3]. Following the epidemic of obesity due to the consumption of high-calorie diet and sedentary lifestyle, NAFLD is becoming a serious global health problem, representing the leading cause of liver disease [4,5,6]. NAFLD defines a wide pathological spectrum, ranging from indolent liver fat storage, which may remain uncomplicated to nonalcoholic steatohepatitis (NASH) characterized by hepatocellular damage, lobular necroinflammation, and the activation of fibrogenesis [7,8]. NASH potentially predisposes to liver cirrhosis and hepatocellular carcinoma (HCC), and is projected to become the first cause of liver-related morbidity and mortality in Western countries within ten years [9].

A growing body of evidence indicated that NAFLD develops as a result of a complex interaction between genetic susceptibility and other environmental factors [10], typically hypercaloric diet and physical inactivity. Furthermore, NAFLD development and progression is modulated by epigenetic factors such as liver-specific DNA-methylation and microRNAs, which regulate the liver transcriptome. There is currently no drug therapy for the treatment of NAFLD, and the mainstay management of the disease is lifestyle modification. The interface between the nutritional environment and cellular/genetic processes is being referred to as nutritional genomics or “nutrigenomics” [11]. In the last years, nutrigenomics has contributed to the understanding of how nutrition affects the switch from health to disease by modulating the expression of an individual’s genetic makeup.

In this review, we focus upon the interaction of genetic background and diet with regard to the development and progression of NAFLD, which can be considered a good candidate for nutrigenomic studies. Indeed, individuals vary in their nutrients requirement and response to diet according to their genetic features, suggesting that personalized nutrition may represent an individualistic therapeutic approach to the disease, with risk being modulated by nutritional, lifestyle, and genetic influences.

## 2. Pathophysiology of NAFLD

Hepatic fat accumulation results from an imbalance between the uptake and synthesis of fatty acids on one hand, and their utilization and secretion on the other hand. Esterification in the form of triglycerides (TGs) in hepatocellular lipid droplets represents the safest way to store fatty acids in the liver [2]. Excess hepatocellular TG derives from several sources, including dietary fatty acids, increased peripheral lipolysis due to adipose tissue insulin resistance, and elevated hepatic de novo lipogenesis (DNL) due to hyperinsulinemia [10,12,13]. A relative reduction of lipid secretion through very-low-density lipoproteins (VLDL) and of mitochondrial oxidation with the advent of oxidative damage are also involved in hepatic fat accumulation [13].

The multiple hit hypothesis proposed that TG accumulation sensitizes the liver to second insults, which are represented by (a) direct lipotoxicity and endoplasmic reticulum stress, (b) hepatocellular oxidative stress secondary to free radicals produced during β- and ω-oxidation of free fatty acids (FFAs), (c) inflammation triggered by endotoxin engaging Toll-like receptor-4 in Kupffer cells and hepatocytes due to increased intestinal permeability and to qualitative and quantitative changes in gut microbiota [14,15], (d) insulin resistance and altered profile of adipokines [16], (e) activation and senescence of hepatic stellate cells [17]. All these conditions lead to hepatic inflammation, cellular damage, and the activation of fibrogenesis. However, other organs (e.g., the adipose tissue, muscle, and intestine) are involved in the pathogenesis of NAFLD, which can thus be defined as a systemic metabolic disorder [18].

## 3. Genetics of NAFLD

Although many individuals share environmental risk factors such as obesity, not all at risk subjects develop fatty liver, suggesting that the genetic background plays a significant role in determining this condition. Epidemiological, familial, and twin studies [19,20] support a strong heritability component in NAFLD and NASH [21]. Indeed, there is a huge inter-ethnic variability in the predisposition towards NAFLD [4].

The rs738409 C > G variant in patatin-like phospholipase domain-containing 3 (PNPLA3)—encoding for the amino acid substitution I148M—has been identified as a major determinant of hepatic fat content [22,23]. The size effect of the I148M variant on the risk of NAFLD is the strongest ever reported for a common variant, modifying the genetic susceptibility of NAFLD [24,25] and also determining liver disease severity and the progression of NAFLD [23,24,26]. The polymorphism exerts its detrimental activity by leading to PNPLA3 accumulation at the surface of hepatocellular lipid droplets, where it likely inhibits the activity of other lipases, determining reduced TG turnover and dismissal [27]. Accumulation of the mutated I148M protein is triggered by acquired factors. Indeed, PNPLA3 is nutritionally regulated at the transcriptional level in response to increased insulin levels by sterol regulatory element-binding protein (SREBP)-1c through carbohydrate-mediated activation of liver X receptor/retinoid X receptor (LXR/RXR). Furthermore, fatty acids can inhibit PNPLA3 protein degradation [28,29,30]. As a consequence, a significant interaction exists between the effect of adiposity on the phenotypic expression of the PNPLA3 mutation and the risk of NAFLD and cirrhosis [31]. This represents a clear demonstration of a gene ↔* environment interaction in the pathogenesis of a complex disease.

The rs58542926 C > T genetic variant of the transmembrane 6 superfamily member 2 gene (TM6SF2), encoding the E167K variant, is another determinant of hepatic triglyceride content [32] and the full spectrum of liver damage linked to hepatic fat accumulation, including NASH, necroinflammation, and fibrosis. The protein is involved in VLDL lipidation and secretion, and the mechanism of the association is related to the retention of lipids within intracellular lipid droplets [32,33].

Variation in the GCKR (glucokinase regulatory gene) locus has been associated with fasting triglycerides levels [34,35,36,37] and NAFLD [38,39,40,41]. GCKR regulates DNL by controlling the influx of glucose in hepatocytes. A common missense loss-of-function GCKR mutation (rs1260326), encoding for the P446L protein variant, may represent the causal variant underlying the association with hepatic fat accumulation [37,42,43]. The P446L variant indeed affects GCKR’s ability to negatively regulate glucokinase in response to fructose-6-phosphate, thereby determining constitutive activation of hepatic glucose uptake [39]. This leads to decreased circulating fasting glucose and insulin levels, but on the other hand it would lead to increased glycolysis and the production of malonyl-CoA which favors hepatic fat accumulation by serving as a substrate for lipogenesis and by blocking fatty acid oxidation.

The rs641738 C > T variant linked to the 3’ untranslated region of the MBOAT7 (membrane bound *O*-acyltransferase domain-containing 7 gene) was recently associated with the risk of NAFLD, inflammation, and fibrosis by reducing protein expression and phosphatidyl-inositol desaturation in hepatocytes [44].

Finally, case–control studies demonstrated a role of other genetic variants implicated in inflammation, insulin signaling, oxidative stress, and fibrogenesis in NAFLD progression (reviewed in [45]).

## 4. Nutrients and NAFLD

Diet is an important contributor to the pathogenesis of NAFLD. Excessive energy intake results in excess adiposity, insulin resistance, and the consequent inappropriate release of fatty acids into the circulation. Dietary fat represents the primary source of adipose tissue fatty acids, which are subsequently released and re-esterified in the liver [46]. The composition of diet and the nature of fatty acids may also impact hepatic DNL by directly regulating the expression of genes involved in fatty acid synthesis. It has been established that—at least in animal models—polyunsaturated fatty acids (PUFAs) down-regulate sterol regulatory element binding protein-1c (SREBP-1c) [47], whereas saturated fatty acids (SFAs) stimulate its expression.

NAFLD patients tend to have a dietary pattern characterized by a higher consumption of SFAs, cholesterol, and fructose, and lower ingestion of PUFAs and antioxidants (vitamin C and E) [48]. It is possible that altering dietary macronutrient composition, which addresses specific molecular pathways and modifies gene and protein expression, modulates the clinical outcome.

### 4.1. Saturated and Unsaturated Fatty Acids

The consumption of diets rich in saturated fatty acids triggers DNL. The action of SFAs on hepatic lipid metabolism is influenced by the peroxisome proliferator-activated receptor (PPAR)-γ coactivator-1beta (PGC-1β). A high-fat diet enriched with SFAs stimulates liver PGC-1β, which coactivates SREBP-1c and enhances its transcriptional activity in lipogenic genes, such as stearoyl-CoA desaturase-1 (SCD-1), fatty acid synthase (FAS), and diacylglycerol acyltransferase (DGAT) [49]. Dietary patterns enriched in SFAs have also been associated with a higher expression of the unfolded protein response (UPR)-related components, all elicited by endoplasmic reticulum stress. Moreover, an increase in caspase 3 activity was also observed [50].

The ingestion of monounsaturated fatty acids (MUFAs) lowers cardiovascular risk and improves lipid profile. Replacement of SFAs with MUFAs ameliorates serum glucose levels and blood pressure [51]. MUFAs inhibit the oxidation of LDL-cholesterol and decrease the serum level of triglycerides by activation of peroxisome proliferator-activated receptor α (PPARα). PUFAs—in particular docosahexaenoic acid (DHA)—are long-chain n-3 fatty acids that reduce TG accumulation and improve hepatic steatosis [52]. PUFAs might prevent NAFLD by activation of peroxisome proliferator-activated receptors (PPARs) and inhibition of SREBP-1c. PPARs are the most extensively characterized nuclear receptors that are regulated by fatty acids [53]. There are three major isoforms of PPARs: PPARα, PPARβ, and PPARγ, with PPARα being the predominant isoform in liver. Ligand activation of PPARα is associated with transcriptional up-regulation of a wide range of genes for proteins associated with fatty acid oxidation and lipoprotein metabolism [52].

### 4.2. Carbohydrates

Carbohydrates are the main substrate that drive DNL in the liver and the adipose tissue. In particular, the intake of sucrose-sweetened beverages has been associated with a higher amount of visceral adipose tissue (VAT) and liver fat accumulation compared with milk, diet cola, and water [54]. A study which compared the consumption of fructose-sweetened with glucose-sweetened beverages revealed that mainly fructose-containing drinks increase visceral adipose volume, promote DNL, and decrease insulin sensitivity in overweight/obese subjects [55]. Fructose consumption induces hepatic lipid accumulation by (a) activation of lipogenic gene expression and (b) direct flow of fructose carbon into the glycolytic pathway [56]. Fructose but not glucose ingestion activates hepatic carbohydrate-responsive element-binding protein (ChREBP), which regulates glycolytic and lipogenic gene expression programs [57]. Moreover, dietary fructose impairs the insulin signaling pathway by increasing c-Jun N-terminal kinase (JNK) activity and insulin receptor substrate 1 (IRS-1) phosphorylation [58]. Finally, elevated dietary fructose intake facilitates bacterial overgrowth in the small intestine, accompanied by heightened intestinal permeability. It increases endotoxin levels in the portal vein and contributes to the mechanism of NAFLD [59].

### 4.3. Proteins

Although the effects of dietary protein on hepatic lipids is not clear, clinical trials show that protein intake has a beneficial impact on the course of NAFLD [60]. Experiments in rodents demonstrated that a high-protein and low-carbohydrate diet reduces adipose tissue deposition, improves glucose homeostasis, and decreases steatosis by inhibition of DNL [61]. Dietary protein has a beneficial effect on glucose metabolism and decreases insulin output. Protein intake is essential for the regeneration of hepatocytes and supplies crucial amino acids for the inclusion of fat into lipoproteins for liver export, thus preventing steatosis [62].

Moreover, as amino acid catabolism is an energy-requiring process, a higher protein consumption might trigger hepatic lipid oxidation through an increase in hepatic energy expenditure [63].

### 4.4. Metals and Other Dietary Components

NAFLD is also characterized by a derangement in the homeostasis of metals, possibly reflecting an increased oxidative stress and an inflammation condition. In particular, it has been demonstrated that iron and copper dys-homeostasis can contribute to NAFLD pathogenesis. 

Iron (Fe) is an essential nutrient required for erythropoiesis and multiple cellular metabolic functions. However, an excess of iron may be detrimental, mostly via the formation of reactive oxygen species, which may lead to severe organ damage. Iron perturbations are frequently observed in patients with NAFLD. Hepatic iron overload in conjunction with metabolic syndrome is commonly observed, and has been termed dysmetabolic iron overload syndrome (DIOS). Increased ferritin levels are usually associated with NASH and the severity of liver damage, whereas in patients with mild iron overload, iron depletion may decrease insulin resistance and liver damage [64,65]. Moreover, iron depletion up-regulated glucose uptake and increased insulin receptor expression and signaling in hepatocytes in vitro and in vivo [66], whereas dietary iron supplementation induced insulin resistance and dyslipidemia [67]. To investigate the mechanisms underlying iron accumulation in NAFLD patients, we recently examined the effect of fatty acids on hepatic iron metabolism. The exposure of hepatocytes to FFAs, leading to steatosis, was associated with a subversion of iron metabolism characterized by increased expression of transferrin receptor and the facilitation of iron storage [68].

Copper is known to be essential for many physiologic processes, such as antioxidant defense, lipid peroxidation, and mitochondrial function. A copper (Cu)-restricted diet induced NAFLD in animal models [69]. In addition, hepatic Cu deficiency is observed in human NAFLD and is associated with steatosis, NASH progression, and insulin resistance [70,71]. Additionally, NAFLD patients with low copper levels have increased iron stores [70]. In experimental models, dietary copper deficiency determined increased hepatic iron content as well as steatosis and insulin resistance [71]. Intestine-specific genetic inactivation of high-affinity copper import in mice also resulted in hepatic iron overload [72]. Interestingly, fructose feeding exacerbates complications of copper deficiency. In rats, fructose consumption impaired copper status and precipitated copper deficiency, possibly inhibiting its absorption through the intestinal epithelium. 

Moreover, dietary copper deficiency and fructose feeding work together to exacerbate liver damage and accelerate hepatic fat accumulation [69,73]. Hepatic carnitine palmitoyltransferase 1 CPT-1 expression was significantly inhibited whereas FAS was markedly upregulated [69] in copper-deficient rats fed with fructose. Finally, rats fed either high-sucrose or low-copper diet had increased hepatic inflammation and fibrogenesis and hepatic stellate cell activation, while the combination of dietary factors induced fasting hepatic insulin resistance and liver damage [74].

## 5. Epigenetics in NAFLD

The emerging field of epigenetics, a heritable but reversible phenomenon that allows for fine-tuning gene expression without altering DNA sequence, provides a new perspective on the pathogenesis of NAFLD [75]. The epigenetic modulation of gene expression includes chromatin remodeling (histone modifications), DNA methylation, and regulation of RNA processing, stability, and translation through specific binding of small RNA molecules (microRNAs). Epigenetic modifications are known to be involved in hepatic lipid metabolism, insulin resistance, mitochondrial dysfunction, and oxidative stress—all of which contribute to NAFLD development and progression [76,77]

There has been considerable progress in understanding epigenetic mechanisms by which the fetal liver might be “primed” by gestational over-nutrition, and epigenetic modifications can be inherited from parents to their offspring. For example, increased methylation leading to down-regulation of PGC1α and mitochondrial biogenesis has been reported in patients with NAFLD [78]. In addition, fetal exposure to high fat diet (HFD) has been associated with histone modifications of the phosphoenol pyruvate carboxy kinase 1 gene (*PCK1*), the rate-limiting enzyme in the gluconeogenic pathway. These data suggest that epigenetic mechanisms prime offspring liver for increased hepatic glucose production [79]. Moreover, the livers of offspring mice exposed to maternal HFD were hypomethylated at the Cdkn1a gene, which codes for a cell cycle inhibitory protein, impairing the regenerative capacity and response to damage [80].

DNA methylation is considered a key factor in the process leading from simple steatosis to NASH, and it may be modulated by dietary deficiency of fundamental methyl donors such as betaine, choline, and folate [81]. Supplementation of betaine is associated with a reduced methylation of the promoter of microsomal triglyceride transfer protein (MTTP), involved in VLDL lipidation, consequently promoting TG export from the liver [82], whereas folate deficiency induces triglycerides accumulation in the liver by influencing the expression of genes involved in fatty acids synthesis [83].

Moreover, in a study that included obese patients with NAFLD and healthy controls, NAFLD-specific methylation differences were seen for nine genes coding for key enzymes catalyzing the initial steps in glucose, lipid, acetyl-CoA, and oligosaccharide synthesis and pathway members of insulin signaling. These NAFLD-specific methylation changes were partially reversible after bariatric surgery [84].

## 6. A Role for Microbiota?

Each individual contains about 1.5–2 Kg of micro-organisms (bacteria, fungi, viruses, and bacteriophages) within the gut. The intestinal microbiota contains >100-fold genes than the human genome, which are responsible for the production and modifications of a wide range of circulating metabolites, regulating the function of organs (reviewed in [85]). Human diseases are associated with modification of the human microbiota, and the diet is able to profoundly reshape the microbiotal composition within hours. It has already been demonstrated that the beneficial effect of some drugs (e.g., metformin) is partly mediated by their effect on the microbiota [86]. Therefore, it is likely that the effect of diet on the risk of NAFLD is partly mediated by alterations of the microbiota. 

Intake of alcohol or a diet rich in saturated fatty acids, cholesterol, and fructose impairs the intestinal barrier by weakening the mucus-associated defense, impairing tight junction function and causing intestinal inflammation with subsequent translocation of bacterial pathogens into the bloodstream and to the liver. Several lines of evidence suggest that endotoxemia is involved in the pathogenesis of NAFLD/NASH. Endotoxin induces systemic inflammation and hepatic necroinflammation mainly through its action on toll-like receptor 4 (TLR4), and NAFLD/NASH patients have increased endotoxin levels in the blood. Indeed, patients with NAFLD have significantly increased gut permeability [15] and a higher prevalence of small intestine bacterial overgrowth compared to healthy controls, which in turn increase the absorption of endotoxin. Specifically, a dysbiotic microbiome is often observed among obese individuals and is considered to be one of the major risk factors for NAFLD [87]. Both obesity and NAFLD are associated with a higher proportion of Gram-negative bacterial species in the gut microbiome, and microbial populations of NASH patients have been suggested to have a higher ability to produce alcohol [88]. Given the association between specific microbial population and NAFLD, the development of a specific metabolic diagnostic profile could possibly represent a non-invasive therapeutic approach [89,90], and may be also useful for monitoring fibrosis stage.

## 7. Interaction between Genetic and Nutritional Factors

**Figure 1:**
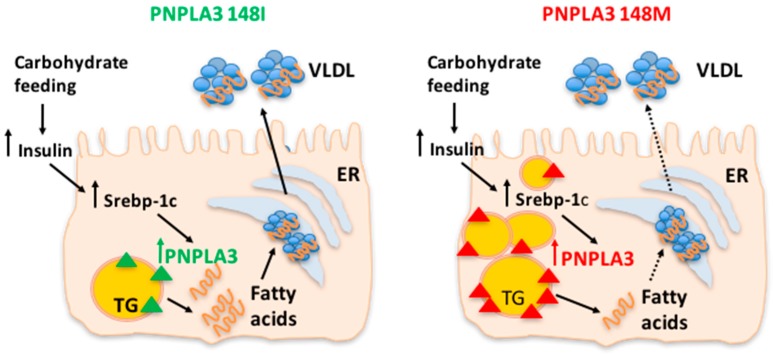
The patatin-like phospholipase domain-containing 3 (PNPLA3) 148M variant favors triglyceride (TG) accumulation upon carbohydrate feeding. The reduced capacity of subjects with the PNPLA3 148M/M genotype to hydrolyze TG in the liver is exacerbated by high dietary sugar intake because carbohydrate-mediated up-regulation of PNPLA3 would favor the accumulation of the pathological protein on the surface of lipid droplets. Personalized dietary intervention based on reduction of carbohydrates intake in individuals with the PNPLA3 at risk genotype may lead to more effective clinical outcomes for NAFLD patients. ER: endoplasmic reticulum; VLDL: very low-density lipoprotein. Solid lines indicate unmodified pathways, dashed lines indicate pathways which are reduced.

The PNPLA3 I148M variant represents a first and until now almost unique example of a genetic factor for which a clear interaction between the gene and environment has been robustly demonstrated. The PNPLA3 I148M variant interacts with environmental stressors such as obesity [31,91] and alcohol consumption [92], which induce fatty liver. Similarly, individuals who carry the I148M variant are more susceptible to increased fat content when dietary carbohydrate intake—specifically sugar—is high [93]. A nutrigenetic analysis with the I148M variant in Hispanic children revealed that hepatic TG accumulation was related to carbohydrate and sugar intake in the homozygous 148M/M group, whereas no effect was observed in I/I and I/M individuals. In Italian adolescents, the 148M variant interacted with dietary intake of fructose-sweetened beverages in determining NAFLD [94,95]. These results are consistent with the notion that the reduced capacity of subjects with the 148M/M genotype to hydrolyze TG in the liver would be exacerbated in the context of high dietary sugar, because carbohydrate-mediated up-regulation of PNPLA3 would favor the accumulation of the pathological protein on the surface of lipid droplets, resulting in the inhibition of other lipases, mainly PNPLA2 (Figure 1). Consistently, a short-term dietary intervention study provided evidence that a hypocaloric and low-carbohydrate diet reduces hepatic fat to a greater extent in PNPLA3 148M/M carriers as compared to wild-type individuals, despite similar weight loss in both genotype groups.

In addition, hepatic fat accumulation could be modulated by the interaction between PNPLA3 I148M and dietary omega 6/omega 3 PUFA [96]. The I148M variant has decreased activity in hydrolyzing the n-9 fatty acids [97]. The n-9 represent the most common fatty acids in the diet, but they are also synthesized starting from n-6. It could be speculated that the newly-synthetized TGs tend to accumulate in the liver in patients carrying the 148M risk variant, leading to steatosis. In the meantime, the excess of n-6 not incorporated in TG will be utilized as substrate for the synthesis of proinflammatory species, triggering NASH [96]. Recently, data from the WELCOME trial revealed that in patients with NAFLD the PNPLA3 148M/M genotype was associated with higher liver fat percentage and lower DHA tissue enrichment and reduced amelioration of aminotransferases after DHA+EPA supplementation for 15–18 months [98]. Consistently, in a randomized trial of short-term DHA supplementation in children with NAFLD, carriage of the 148M risk variant reduced the beneficial effect on liver enzymes and circulating lipids [99]. These results suggest that PNPLA3 I148M is involved in omega-3 fatty acids mobilization in the liver, and subjects with PNPLA3 148M/M genotype have lower levels of DHA [96]. Additionally, omega-3 fatty acids decrease the expression of SREBP1c [100], and carriers of the PNPLA3 148M allele tend to have decreased DNL as a feed-back mechanism to avoid excessive lipid accumulation [101]. Thus, it is possible that the lack of response to omega-3 fatty acids supplementation in decreasing liver fat percentage in subjects with PNPLA3 I148M/M is explained by the fact that these subjects already have low levels of DNL.

### GCKR

Initial evidence has been gathered on the interaction between GCKR variation and diet in the pathogenesis of dyslipidemia, although no data are yet available specifically for NAFLD. GCKR variation determines higher fasting TG levels and higher absolute plasma postprandial TG and VLDL-cholesterol levels following a fat-meal challenge [102,103]. Supporting the presence of gene–diet interaction, in metabolic syndrome individuals participating to the LIPGENE study it has been reported a statistically significant association between GCKR rs1260326 polymorphism and plasma omega-3 fatty acids modulating insulin resistance and inflammatory markers. Moreover, an interaction has been demonstrated between the P446L GCKR variant and Mediterranean diet on circulating TG concentrations. Indeed, adherence to this dietary pattern blunted the negative effect of the risk 446L on circulating TG, likely reflecting the activation of DNL [104]. Consistent with these latter findings, the influence of GCKR genotype on serum cholesterol was reported to depend on the MUFA: SFA ratio in the diet [105].

## 8. Future Perspectives

The field is clearly only in its early stages, and in order to develop a full nutrigenomic approach to the management of NAFLD, key information is still missing.

In particular, large well-characterized cohorts with contemporary determination of (a) nutritional (b) genomics (and possibly epigenetics, transcriptomics, and proteomics) (c) microbiota composition (d) hepatic fat and liver damage assessment will be needed together with long-term follow-up of patients. The integration of this information could be used to define biomarkers and to develop tools to calculate a NAFLD risk score by changing our ability to control or cure the disease.

To apply these findings to the treatment of patients with NAFLD, randomized controlled trials aimed at the study of the interaction between diet and genetic/epigenetic factors on health-related outcomes will have to be conducted, in order to optimize the individual’s response to the intervention.

## 9. Conclusions

**Figure 2:**
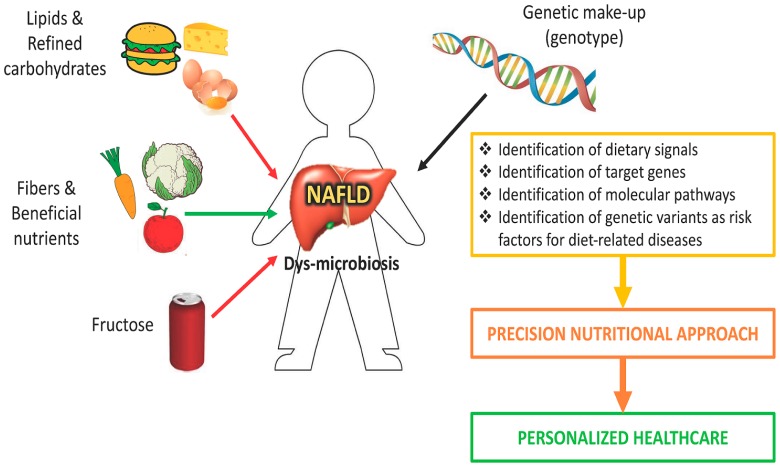
A nutrigenomic approach for nonalcoholic fatty liver disease (NAFLD). Dietary intervention based on knowledge of nutritional requirement, nutritional status, and genetic makeup (personalized medicine) can be used in the management of NAFLD patients.

NAFLD is becoming a serious global health problem, representing the leading cause of liver disease. To date there are no approved drugs for the treatment of NAFLD, and the main clinical recommendation as an initial step is lifestyle modification, that is reduced caloric intake and physical exercise. Supporting a key role of gene–diet interaction in NAFLD development, it is important to understand how dietary composition, which influences specific molecular pathways and modifies gene and protein expression, could modulate the clinical outcome. Personalized dietary intervention based on the reduction of carbohydrates and sugar intake in genetically predisposed individuals with the PNPLA3 at-risk genotype may lead to more effective clinical outcomes for NAFLD patients. Since an individual’s genetic makeup influences how nutrients are assimilated, stored and excreted, personalized nutrition which takes into account the genetic features of patients could have immediate potential for clinical translation, representing an individualized therapeutic approach to the disease (Figure 2).

## Abbreviations

NAFLD	Nonalcoholic fatty liver disease
NASH	Nonalcoholic steatohepatitis
TG	Triglycerides
DNL	De novo lipogenesis
VLDL	Very low-density lipoproteins
FFAs	Free fatty acids
PNPLA3	Patatin-like phospholipase domain-containing 3
SREBP	Sterol regulatory element-binding protein
LXR	Liver X receptor
RXR	Retinoid X receptor
TM6SF2	Transmembrane 6 superfamily member 2
GCKR	Glucokinase regulator
MBOAT7	Membrane bound O-acyltransferase domain-containing 7
PUFAs	Polyunsaturated fatty acids
SFAs	Saturated fatty acids
MUFAs	Monounsaturated fatty acids
PGC-1	PPAR-γ coactivator-1beta
SCD1	Stearoyl-CoA desaturase-1
FAS	Fatty acid synthase
DGAT	Diacylglycerol acyltransferase DGAT
PPARs	Peroxisome proliferator-activated receptors
ChREBP	Carbohydrate-Responsive Element-Binding Protein
JNK	c-Jun N-terminal kinases
IRS-1	Insulin receptor substrate 1
Fe	Iron
Cu	Copper
Cdkn1a	Cyclin-dependent kinase inhibitor 1a
PCK1	Pyruvate carboxy kinase 1 gene
MTTP	Microsomal triglyceride transfer protein
SIRT1	Sirtuin 1
